# The Molecular Mechanisms behind Advanced Breast Cancer Metabolism: Warburg Effect, OXPHOS, and Calcium

**DOI:** 10.31083/j.fbl2903099

**Published:** 2024-03-13

**Authors:** Erna Mitaishvili, Hanna Feinsod, Zachary David, Jessica Shpigel, Chelsea Fernandez, Moira Sauane, Columba de la Parra

**Affiliations:** 1 Department of Chemistry, Herbert H. Lehman College, City University of New York, New York, NY 10468, USA; 2 PhD Program in Biology, The Graduate Center, City University of New York, New York, NY 10016, USA; 3 Department of Chemistry, Columbia University, New York, NY 10027, USA; 4 Department of Biological Sciences, Herbert H. Lehman College, City University of New York, New York, NY 10468, USA; 5 PhD Programs in Biochemistry and Chemistry, The Graduate Center, City University of New York, New York, NY 10016, USA

**Keywords:** TNBC, Warburg effect, metabolic reprogramming, glycolysis, hexokinase, OXPHOS, mitochondrial Ca^2+^, ROS, JAK/STAT, miRNAs

## Abstract

Altered metabolism represents a fundamental difference between cancer cells and normal cells. Cancer cells have a unique ability to reprogram their metabolism by deviating their reliance from primarily oxidative phosphorylation (OXPHOS) to glycolysis, in order to support their survival. This metabolic phenotype is referred to as the “Warburg effect” and is associated with an increase in glucose uptake, and a diversion of glycolytic intermediates to alternative pathways that support anabolic processes. These processes include synthesis of nucleic acids, lipids, and proteins, necessary for the rapidly dividing cancer cells, sustaining their growth, proliferation, and capacity for successful metastasis. Triple-negative breast cancer (TNBC) is one of the most aggressive subtypes of breast cancer, with the poorest patient outcome due to its high rate of metastasis. TNBC is characterized by elevated glycolysis and in certain instances, low OXPHOS. This metabolic dysregulation is linked to chemotherapeutic resistance in TNBC research models and patient samples. There is more than a single mechanism by which this metabolic switch occurs and here, we review the current knowledge of relevant molecular mechanisms involved in advanced breast cancer metabolism, focusing on TNBC. These mechanisms include the Warburg effect, glycolytic adaptations, microRNA regulation, mitochondrial involvement, mitochondrial calcium signaling, and a more recent player in metabolic regulation, JAK/STAT signaling. In addition, we explore some of the drugs and compounds targeting cancer metabolic reprogramming. Research on these mechanisms is highly promising and could ultimately offer new opportunities for the development of innovative therapies to treat advanced breast cancer characterized by dysregulated metabolism.

## 1. Introduction

Breast cancer is a significant global health problem and is the second most commonly diagnosed cancer world-wide, affecting over 1.6 million women annually with a 20% mortality rate [1,2]. Most breast cancers can be broadly categorized as invasive or non-invasive and then further categorized by molecular features such as the presence or absence of hormone receptors and growth factor proteins. Among the invasive subtypes of breast cancer, triple-negative breast cancer (TNBC) is recognized as a particularly aggressive form, representing 10–15% of all breast cancer occurrences in women. TNBC is given the name due to its three-fold deficiency in the estrogen receptor (ER), progesterone receptor (PR), and human epidermal growth factor receptor 2 (HER2), which are considered the main treatment targets in breast cancer patients. While other breast cancer subtypes have the potential of targeted treatment, patients with TNBC lack targetable receptors, resulting in limited treatment options and an overall worse prognosis. TNBC patients have a significantly higher incidence of metastasis, which is still considered the predominant cause of patient mortality. Upon metastasis, the cancer becomes increasingly more difficult to treat due to tumor heterogeneity and inability for physical intervention by surgery, creating more obstacles for clinicians as the cancer becomes more complex. At the cellular level, the success of the metastasis requires the tumor cells to adapt and exploit the stressful tumor microenvironment to acquire the necessary nutrients to survive the migration process and thrive at the new site. One of these adaptations is a cancer cell’s ability to reprogram its metabolism, using a variety of bioenergetic pathways to produce metabolic intermediates, amino acids precursors, nucleotides, lipids, and sugars to be able to successfully fuel the energetic demands of cellular proliferation. For this reason, metabolic reprogramming in cancer has been thoroughly validated and is now considered a defining hallmark of cancer [3]. This review examines a select number of molecular mechanisms, within the context of two major metabolic pathways—glycolysis and oxidative phosphorylation (OXPHOS)—that play a crucial role in TNBC progression. These mechanisms include the Warburg effect, glycolytic adaptations, microRNA regulation, mitochondrial involvement, mitochondrial calcium signaling and the emerging role of JAK/STAT signaling. Potential drug targets for some of these mechanisms that may offer new therapeutic interventions are also explored.

## 2. The Warburg Effect

**Fig. 1.**
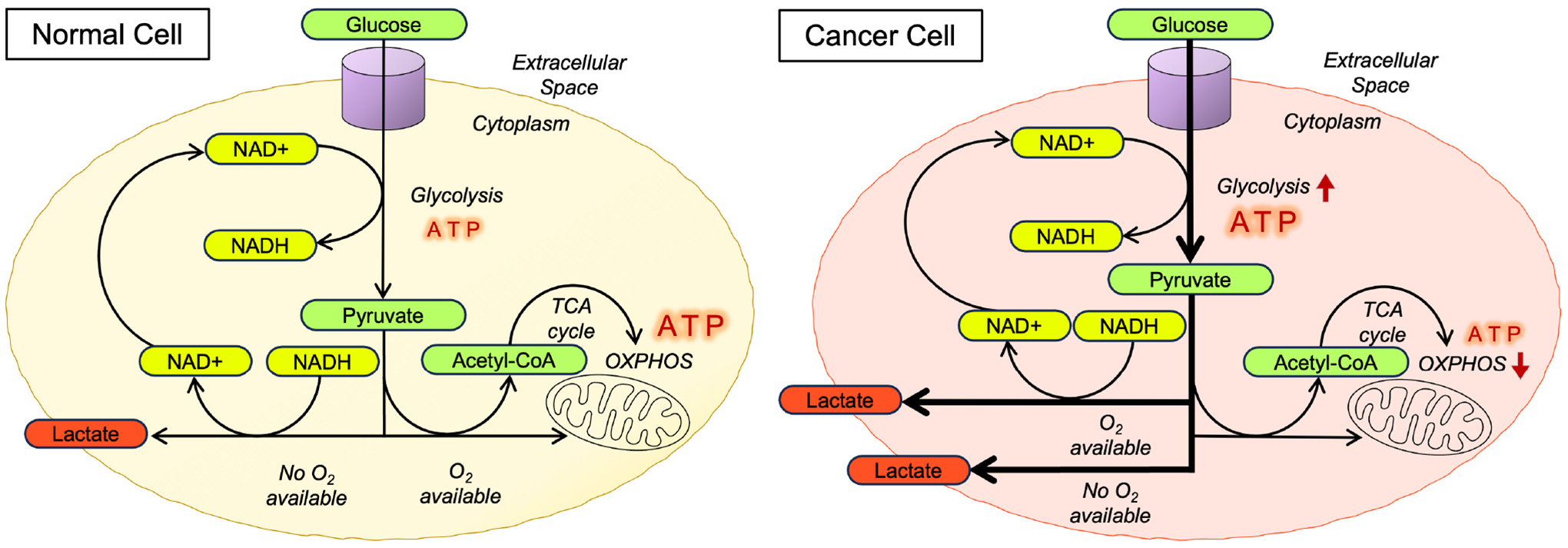
Schematic representing the differences observed in glucose metabolism between normal cells and cancer cells. In normal cells, glucose that enters the cells undergoes glycolysis to generate pyruvate, nicotinamide adenine dinucleotide (NAD+), and a modest amount of adenosine triphosphate (ATP). When O_2_ is available, pyruvate gets converted to Acetyl-CoA in the mitochondria, entering the tricarboxylic acid (TCA) cycle and cellular respiration continues through to oxidative phosphorylation (OXPHOS), generating an abundant amount of ATP. However, if O_2_ is unavailable, the cell will direct pyruvate to generate lactate, allowing the regeneration of NAD+ to cycle back into glycolysis. In the Warburg effect or aerobic glycolysis, cancer cells will preferentially generate lactate from glucose, regardless of the availability of O_2_ in the cell, rather than prioritizing the production of ATP through OXPHOS. The continuous regeneration of NAD+ helps it loop back and resupply glycolysis.

In the 1920s, Otto Heinrich Warburg made a ground-breaking discovery: cancer cells, even in oxygen-rich environments, preferentially metabolize glucose to lactate by-passing the more efficient OXPHOS pathway [4–6]. This phenomenon, suitably known as the Warburg effect (also referred to as aerobic glycolysis), revolutionized our understanding of cancer metabolism (Fig. 1) [4]. In the presence of oxygen (O_2_) or aerobic conditions, noncancerous cells utilize glucose metabolism efficiently and sequentially by employing glycolysis, a low energy yield pathway, as a precursor to a higher energy yield pathway, oxidative phosphorylation (OXPHOS). During glycolysis, glucose transforms into pyruvate and carbon dioxide (CO_2_), generating adenosine triphosphate (ATP) and nicotinamide adenine dinucleotide (NADH) from nicotinamide adenine dinucleotide (NAD+). The generated NADH then becomes a critical energy input for OXPHOS, the cell’s primary ATP production source and simultaneously recycles back into NAD+ to sustain glycolysis. However, when there is a lack of O_2_, or anaerobic conditions, cells will adopt an alternative metabolic route. In this scenario, glycolysis-derived pyruvate is converted to lactate, to ensure a continuous supply of NAD+ for glycolysis, resulting in a less efficient mode of ATP production compared to OXPHOS (Fig. 1). Cancer cells, however, will preferentially generate lactate irrespective of O_2_ availability as a favorable metabolic adaptation (Fig. 1). This altered metabolism or metabolic reprogramming is a fundamental feature in cancer cells and is one of the numerous attributes that distinguish them from a normal cell [3,7].

### The Warburg Effect and the Tumor Microenvironment (TME)

The Warburg effect was initially thought to be a way for cancers to compensate their energy production because the mitochondria were damaged or dysfunctional. Numerous research has challenged this notion, revealing a far more complex scenario with mitochondrial defects not always being at the forefront of this phenotype [4–6]. Some studies have shown mutations in mitochondrial enzymes or mitochondrial DNA as possible contributors to the Warburg effect, however, dysregulation of signaling pathways, activation of certain oncogenes, mutations in tumor suppressors and the tumor microenvironment (TME) appear to be the major contributors on the metabolic reprogramming of cancer [7–10]. As these intricate mechanisms continue to be explored, it is acknowledged that the observed metabolic phenotype differences vary not only across cancer types but by the degree of malignancy, with more aggressive and invasive cancers generally exhibiting elevated glycolytic fluxes [11–14]. This metabolic adaptation that favors glycolysis, leads to the production of various glucose byproducts linked to different metabolic pathways, such as the pentose phosphate pathway (PPP), and the synthesis of essential biomolecules including nucleotides, amino acids, and lipids. Similar adaptations can be observed in stem cells or cells undergoing development, because it makes nutrient acquisition more efficient for new cell production [15]. It is proposed that the TME, a network composed of the tumor cells, immune cells, blood vessels, the extracellular matrix, and other, non-tumor cell types, plays a role in selecting and sustaining this altered metabolism. Lactate generated from aerobic glycolysis creates a highly acidic TME that supports metabolic flexibility and genomic instability [16,17]. A phenomenon known as the “reverse-Warburg effect” discerns the transfer of lactate (and other metabolites) between stromal cells and cancer cells, further contributing to cancer cells exploiting the TME for metabolic adaption [18–20]. Moreover, as tumors outgrow their blood supply’s capacity, they favor oxygen-independent energy production, stabilizing the transcription factor Hypoxia-Inducible Factor 1-alpha (HIF-1*α*), which orchestrates a response that includes increased expression of glycolytic enzymes and glucose transporters [21,22]. This distinctive adaptation to oxygen-independent energy production is often accompanied by the upregulation of vascular endothelial growth factor (VEGF), a key player in promoting angiogenesis, the physiological process through which new blood vessels develop from pre-existing vessels. In hypoxic tumor environments, the activation HIF-1*α* triggers increased VEGF expression, stimulating the formation of new blood vessels [23,24]. This angiogenic response is crucial for sustaining the nutritional and oxygen requirements of rapidly proliferating tumor cells. Tumor cells that rely on glycolysis are capable of surviving in a consistent hypoxic environment. Hypoxia further induces matrix remodeling, influencing the metabolic profile of TNBC cells [14]. The extent to which breast cancer cells exhibit the Warburg effect and utilize OXPHOS can indeed vary significantly among distinct subtypes of breast cancer. Specific types of breast cancer, including invasive status, are presented in Table 1 (Ref. [25–30]), along with their corresponding trends in glycolysis and OXPHOS status.

## 3. Glycolytic Enzymes and Oncogenic Signaling

**Fig. 2.**
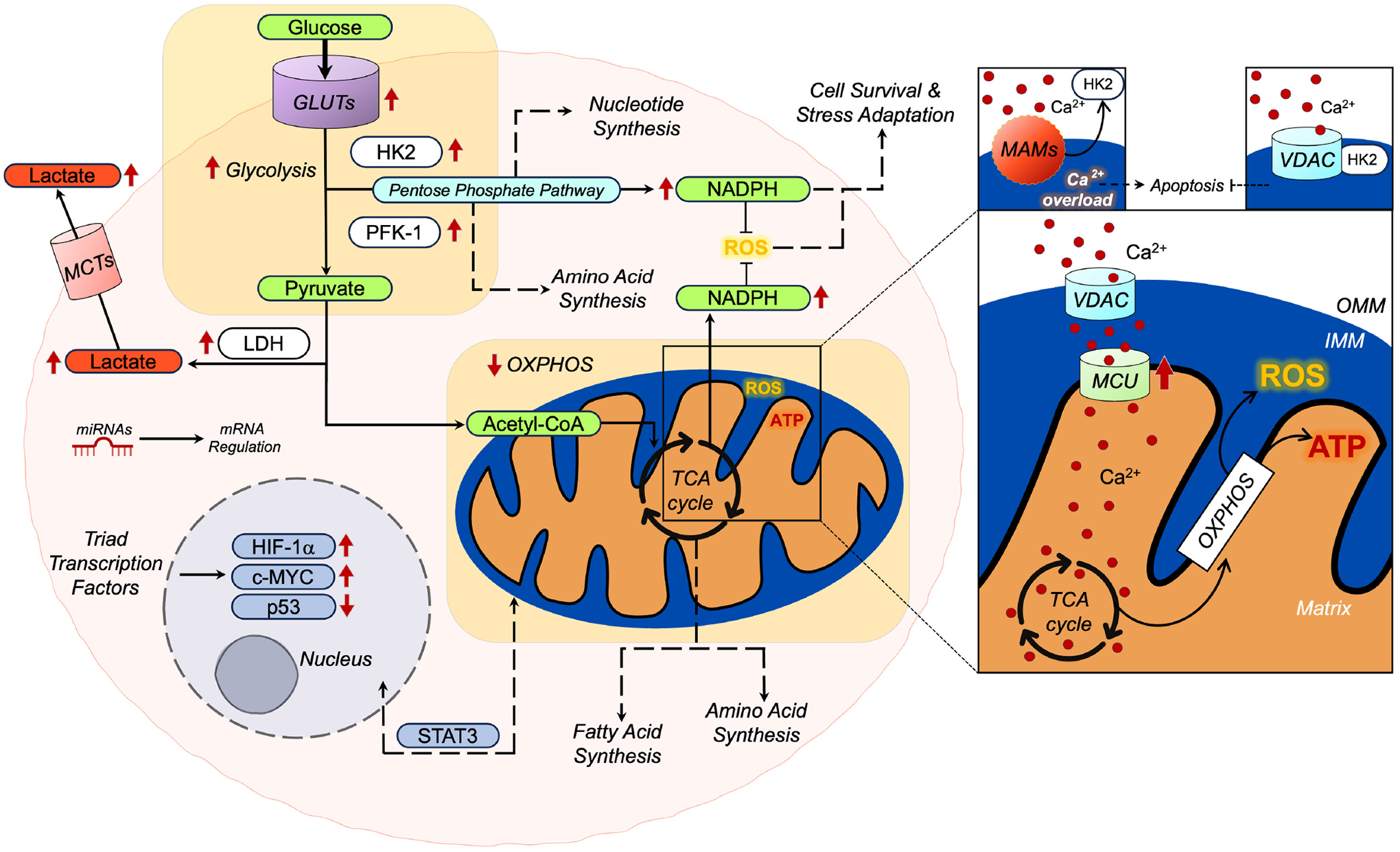
Diagram of the key metabolic features and mechanisms influencing the metabolic reprogramming of triple-negative breast cancer (TNBC). Overexpression of glucose transporters (GLUTs) increases glucose amount and flux into the cell. TNBC cells preferentially use glycolysis for fueling alternative metabolic pathways that are necessary for building cellular biomass and continue proliferation (dashed arrows). Nicotinamide adenine dinucleotide phosphate (NADPH) can be generated through different pathways in the cell and two of those are shown here-the pentose phosphate pathway (PPP) cycle and the tricarboxylic acid (TCA) cycle. NADPH is crucial in maintaining redox homeostasis by acting as an antioxidant to inhibit surplus reactive oxygen species (ROS) damaging the cell. Moderate amounts of ROS are necessary for cancer cell survival and stress adaptation. Gene regulation occurs in the cell through microRNAs (miRNAs), which are shown here in the cytoplasm, regulating gene expression post-transcriptionally of their target mRNA. The triad of transcription factors (TFs) are shown in the nucleus with their relative expressions in TNBC. STAT3, a recognized transcription factor can function canonically or non-canonically by localizing to the mitochondria. Focusing on the diagram of the mitochondria, the Ca^2+^ entering the mitochondria supports mitochondrial metabolism by the regulation of certain TCA cycle enzymes. A simplified schematic shows how hexokinase 2 (HK2) interacts with the mitochondria at the outer mitochondrial membrane (OMM) by binding voltage-dependent anion channel (VDAC). This interaction can inhibit the release of pro-apoptotic factors and inhibit apoptosis (right). On the left, HK2 release from the mitochondria-associated-membranes (MAMs) (endoplasmic reticulum not shown) causes an influx of Ca^2+^ in the mitochondria, causing an overload and leading to cell death. Red arrows (up/down) represent the current knowledge of the expression patterns of the pathway/protein/metabolite. Dashed arrows represent alternative pathways. Monocarboxylate transporters (MCTs), lactate dehydrogenase (LDH), phosphofructokinase-1 (PFK-1) and the mitochondrial calcium uniporter (MCU) are generally upgregulated in TNBC.

Glucose is a primary source of cellular energy and is a central participant in diverse metabolic pathways. Transporters facilitate its cellular entry, leading to glycolysis and the eventual production of pyruvate. In comparison to other subtypes of breast cancer, TNBC displays an elevated dependency on glycolysis [25]. This is evident through increased glucose uptake and lactate secretion, accompanied by the upregulation of essential glycolytic enzymes and transporters, including glucose transporters (GLUTs), hexokinases (HKs), phosphofructokinase-1 (PFK), lactate dehydrogenase (LDH), and monocarboxylate transporter (MCT), as illustrated in Fig. 2. The “triad” of transcription factors, which have been extensively studied and are associated with the glycolytic phenotype in cancer, consist of HIF-1*α*, proto-oncogene *c-MYC*, and tumor suppressor p53 (Fig. 2) [31]. The expression of all three transcription factors can vary and this plays a role in regulating glucose metabolism and the dysregulation of key enzymes and transporters contributing to the processes described below.

### 3.1 GLUT (Glucose Transporter) Proteins

GLUT proteins, encoded by solute carrier 2 (*SLC2*) genes, belong to the Major Facilitator Superfamily (MFS) of membrane transport proteins and serve as the primary glucose transporters in mammalian cells. In humans, there are fourteen GLUT proteins (GLUTs 1–14). Notably, GLUT 1–4 have been implicated in various malignant tumors, including TNBC [32]. A comprehensive bioinformatics study using public databases such as ONCOMINE, Kaplan-Meier Plotter, and cBioPortal was carried out on a cohort of breast cancer patients to explore the intricate connection between GLUT1, GLUT3, GLUT4, and clinical prognostic factors. The study concluded that GLUT1 is overexpressed in TNBC patients and therefore has potential as a novel prognostic biomarker [33]. Another study indicated that the combined mRNA expression of *SLC2A1* (GLUT1) and tumor suppressor, Retinoblastoma protein (RB1), may be significantly correlated, suggesting that tissues with high levels of *RB1* mRNA may also exhibit relatively higher levels of *SLC2A1* mRNA in TNBC [34]. *In vitro* studies have demonstrated promising results with specific inhibitors of GLUT proteins in TNBC cell lines. These include the synthetic non-metabolizable glucose analog, 2-deoxy-D-glucose (2-DG), anti-GLUT1 monoclonal antibody, and phytochemicals like resveratrol. These inhibitors have been associated with decreased glucose uptake, increased apoptosis, and enhanced radiation effectiveness [35–38]. Oncogenes such as *Ras* and *MYC* can promote glucose uptake by upregulating HIF-1*α* and induce GLUT expression. The signaling pathway involved in cell proliferation and survival, phosphoinositide 3-kinase (PI3K)/protein kinase B (Akt)/mammalian target of rapamycin (mTOR) or (PI3K/AKT/mTOR), is often dysregulated in breast cancer and is associated with endocrine therapy resistance [39]. In addition, the PI3K/AKT/mTOR pathway significantly influences the upregulation of glycolytic enzymes and GLUTs [40]. The tumor suppressor p53 is mutated in 20–30% of advanced cancers, including TNBC, and inhibits the expression GLUT1, GLUT3, and GLUT4 [41].

### 3.2 Hexokinases (HKs)

When glucose enters the cell to undergo glycolysis, the first step of glycolysis regulates the intracellular glucose transport through glucose phosphorylation by hexokinases (HK1–4). HK1 is the predominant isoform found in many adult differentiated tissues, meanwhile HK2 is more expressed during development [42,43]. *In vitro* and *in vivo* studies have shown that, compared to HK1, HK2 is highly expressed in cancers, particularly in highly glycolytic cancers, as HK2 can partially enhance glucose flux (Fig. 2) [42–45]. Elevated *HK2* mRNA levels in TNBC cells, relative to non-TNBCs, suggest intensified glycolytic activity and extracellular acidification [46,47]. A recent *in vivo* study by Blaha and colleagues [46] uncovered a kinase-independent function of HK2 that contributes to metastasis. HK2 knockdown in 4T1 cells, which are a murine TNBC equivalent cell line, affected the expression of Epithelial-Mesenchymal Transition (EMT) genes and metastasis by reducing SNAIL protein stability, and nuclear localization via Glycogen Synthase Kinase (GSK3*α*/*β*), a key enzyme that regulates glycogen metabolism and plays a role in metastasis. GSK3-resistant SNAIL variants restored metastasis in HK2-deficient 4T1 cells, offering valuable insights for metastasis research and metabolism [46]. Both HK1 and HK2 are unique in that they are able to bind the mitochondria, amplifying the role of these enzymes as metabolic sensors by their physical interaction with mitochondria at the outer mitochondrial membrane (OMM) at the voltage-dependent anion channel (VDAC) [42,48,49]. VDAC is critical for cell metabolism because it is an entry point for a variety of metabolites such as pyruvate and NADH and can transport calcium ions [43,48–52]. By binding VDAC, HK1 and HK2 are able to scavenge mitochondrial ATP and essentially couple glycolysis with OXPHOS, creating a positive feedback loop to maintain the Warburg phenotype [43,48]. HK2 localizing at the mitochondria enables multifaceted functionalities distinct from its canonical enzymatic activity including autophagy modulation and the inhibiting of apoptosis (Fig. 2) [53,54]. The release of HK2 from VDAC can trigger a robust apoptotic signaling cascade. This release is thought to be initiated when GSK3*β*, a kinase regulated by Akt, phosphorylates a putative HK docking site on VDAC, however, the mechanism by which this occurs is still not completely understood and remains a topic of debate [55,56]. Ciscato and colleagues [54] expanded on the knowledge of HK2 binding at the mitochondria in varying cancer models, including TNBC, by showing that HK2 localizes at the Mitochondria-Associated Membranes (MAMs), a contact site between the OMM of the mitochondria and the endoplasmic reticulum *in vivo* and *in vitro*. MAMs tightly regulates numerous cellular processes ranging from lipid homeostasis to calcium homeostasis by the interaction of protein inositol trisphosphate receptors (IP3Rs) at the ER and VDAC at the OMM which are tethered by the chaperone glucose-regulated protein 75 (GRP75) [57]. The group also observed that HK2 dissociating from the MAMs triggers an influx of calcium in the mitochondria, causing an overload and ultimately cell death (Fig. 2).

### 3.3 Phosphofructokinase-1 (PFK)

There are key enzymatic reactions in glycolysis and one of those key steps is when the glycolytic shunt enzyme 6-phosphofructo-2-kinase/fructose-2,6-biphosphatase 4 (PFKFB4) catalyzes and modulates the levels of fructose-2,6-bisphosphate (F26BP). In turn, this affects the activity of the main rate-limiting step in glycolysis: the enzyme phosphofructokinase-1 (PFK-1) [58]. Several studies have suggested that PFKFB4 is upregulated in various cancers, including TNBC, as part of their metabolic adaptation. Elevated PFKFB4 expression can result in increased levels of F26BP which, thereby, enhances the activity of PFK-1. An *in vitro* study by Dai and colleagues [58] revealed a non-canonical role of PFKFB4 of nuclear translocation in response to hypoxia, activating HIF-1*α* and promoting metabolic plasticity in TNBC. Importantly, increased PFKFB4 expression correlated TNBC patients with poorer outcomes [58]. Within the PFKFB4 family, PFKFB3 has emerged as a pivotal figure in tumor glycolysis, carrying substantial implications for both tumor growth and metastasis. Its function is intricately controlled by crucial factors such as HIF-1*α*, AKT and phosphatase and tensin homolog (PTEN) [59]. The expression of PFKFB3 has been shown to be highly correlated with VEGF expression in breast cancer, thereby contributing to angiogenesis and distant metastasis. TNBC patients have significantly higher expression levels of VEGF in tumors compared to non-TNBC patients [60].

### 3.4 Lactate

In anaerobic glycolysis, lactate dehydrogenase (LDH) catalyzes the reversible final step, converting pyruvate into lactate. Accumulation of the metabolic byproduct lactate and the consequent acidification of the extracellular environment is frequently observed in numerous solid tumors [61]. Monocarboxylate transporters (MCTs) facilitate lactate’s transport across cell membranes (Fig. 2). In particular, TNBC exhibits an increased expression of MCT isoforms, notably MCT1 and MCT4, which are crucial for mediating lactate expulsion and the acidification of the TME. Prior *in vitro* and *in vivo* studies have demonstrated that increased lactate production within cancer cells inhibits the immune surveillance of tumors by T-cells and natural killer (NK)- lymphocytes, consequently facilitating tumor proliferation [62]. The prognostic relevance of plasma LDH levels has been investigated in several breast cancer studies which concluded that there is a correlation between high serum LDH levels and poor overall survival (OS) and progression-free survival (PFS) in breast cancer patients [61,63]. Therefore, the notably elevated expression of LDH-A (monomer of the tetramer LDH) in breast cancer tissues could serve as an indicator of the degree of malignancy.

## 4. A New Player in Metabolism Regulation: JAK/STAT Signaling

The Janus kinase/signal transducer and activator of transcription or JAK/STAT signaling pathway is involved in various physiological processes, including immune response, cell growth, differentiation, and apoptosis. This signaling pathway is a crucial communication hub within the cell and dysregulation of JAK/STAT plays an instrumental role in the development and metastasis of breast cancer [64,65]. The activation of the JAK/STAT pathway can occur through a variety of stimuli, including leptin, a growth factor hormone, and cytokines such as interleukin 4 (IL-4), interleukin 6 (IL-6), interleukin 24 (IL-24), and interferon (IFN). IFN signaling has been linked with a lower incidence of metastases in breast cancer patients and it has been suggested that it can be used as a biomarker to measure the response to chemotherapy in TNBC [66]. The JAK/STAT signaling pathway is intricately modulated through the direct interaction with C/EBP*β*, a transcription factor that has previously established association in regulating breast cancer carcinogenesis and is overexpressed in patients with TNBC [67]. In addition, recent *in vivo* studies using patient-derived xenograft (PDX) models of TNBC have reported that JAK2 signaling not only contributes to tumor cell-intrinsic resistance to paclitaxel, a chemotherapy drug used in TNBC treatment, but also contributes to the modification of the TME [68–70].

### STAT3 and Cancer Metabolism

*In vitro* work in the TNBC cells, MDA-MB-231, demonstrated that IL-6 increases the rate of glucose intake and lactate generation by activating STAT3. Furthermore, overexpression of constitutively activate STAT3, was observed in TNBC and HER2+ patient tissues [71,72]. STAT3 is crucial in advanced cancers because its activation can promote glycolysis by activating transcription of HIF-1*α* as well as transcriptionally activating HK2, *in vitro* [73,74] A comprehensive study utilizing *in vivo* and *in vitro* approaches in addition to patient samples, demonstrated that STAT3 mediated immune cell (NK cells) metabolism to resemble that of tumor cells, resembling the Warburg phenotype. This metabolic reprogramming allowed the NK cells to function more optimally against the tumor cells in the TME [75]. Interestingly, numerous STATs (STAT1, STAT2, STAT3, STAT5, and STAT6) have been shown to localize in the mitochondria in a variety of different cell types to regulate mitochondrial function. For instance, *in vitro* studies have shown how post-translational modifications, such as serine/tyrosine phosphorylation and acetylation of STAT3 enable translocation to the mitochondria [76, 77]. One study in the mouse TNBC equivalent 4T1 cells showed that serine phosphorylated mitochondrial STAT3 can decrease apoptosis and promote tumor cell growth by decreasing the amount of reactive oxygen species (ROS) that is released from complex I [78]. Extending on these findings, another group used TNBC cells and reported that when STAT3 is reduced, it lowers the activity of complex I dehydrogenase, which impairs levels of NAD+. This functions as a reverse signal, linking mitochondrial metabolism to changes in nuclear gene expression, inducing antioxidant genes that help maintain redox balance and malignant cell proliferation, survival, and migration [79]. The mechanisms triggering mitochondrial translocation to engage this non-canonical role of STAT3 have to be further investigated to provide clearer insights on how canonical and non-canonical function can be utilized effectively in immunotherapy cancer therapeutics [80].

## 5. MicroRNAs and Metabolic Reprogramming

MicroRNAs (miRNAs) are a group of short, non-coding RNAs composed of about 20–25 nucleotides and make-up anywhere from 1–5% of the human genome [81, 82]. They play an essential role in regulating gene expression at the post-transcriptional level through interactions with the 3*′*-untranslated regions (3*′*-UTRs) of target messenger RNAs (mRNAs) leading to translation inhibition or mRNA degradation [83]. To date, more than 2000 human miRNAs have been identified, and are estimated to regulate more than one third of cellular mRNAs. Accumulating evidence has linked the dysregulated expression patterns of miRNAs to a variety of diseases, such as cancer. Several studies have shown that miRNAs can act either as tumor suppressors or as oncogenes, and that quantifying miRNA expression in cancer may have diagnostic and prognostic implications [84,85]. Malignant phenotypes in TNBC have been shown to be significantly influenced by miRNAs, as they are crucial regulators of various cellular function mRNAs, such as cell division, apoptosis, invasion, and metastasis [86].

### 5.1 MicroRNAs and Metabolism

There are several miRNAs involved in breast cancer, however, here, the focus will be on miRNAs implicated in TNBC metabolism. *In vitro* and *in vivo* studies have identified miRNAs that exhibit significant correlations with the Warburg effect in TNBC including miR-210–3p, miR-181a, miR-767–5p, and miR-155 [87–89]. Some of these miRNAs are associated with glycolytic metabolism, resulting in dysregulation of glucose consumption and lactate production by modulating glucose transporters and metabolic enzymes including HK2, PKM2, and LDH-A [90]. Within this context, the miR-200 group stabilizes phosphoglucose isomerase (PGI), which contributes to cell invasion and metastasis, *in vitro* and miR-210–3p has a pronounced effect on promoting aerobic glycolysis in TNBC. Mechanistically, miR-210–3p was observed to target GPD1L, a glycerol-3-phosphate dehydrogenase 1-like enzyme, thereby pre-serving the stability of HIF-1*α*, and simultaneously repressing the activity of the tumor suppressor p53, *in vitro*, specifically on the TNBC cells, MDA-MB-231 and HS578 [88,90].

### 5.2 MicroRNAs and Cancer Cell Signaling

Evidence demonstrates that miR-155 functions as an oncogenic miRNA (oncomiR) in human breast cancers, playing a crucial role in tumor development. MiR-155 controls the expression of a number of genes involved in differentiation, inflammation, apoptosis, and transcriptional regulation. Experimentally and through bioinformatics, several miR-155 targets (~149) have been identified. These targets include the tumor suppressor PTEN, Forkhead-box protein O3a (FOXO3a), Cyclin-dependent kinase inhibitor p27 (p27), and Casein Kinase-1*α* (CK1*α*) among others. *In vitro* and *in vivo* studies have demonstrated that miR-155 promotes glycolysis in TNBC cells and enhances glucose uptake in xenograft tumors by suppressing miR-143 and activating STAT3 [91]. Additionally, in a study of 50 TNBC patient specimens and PET images, a significant positive correlation was observed between miR-155 expression and glucose utilization in breast tumors. This association was mediated through miR-155 inhibiting the upstream inhibitor of c-MYC, FOXO3a, thereby initiating activation of c-MYC (PIK3R1-PDK/AKT-FOXO3a-cMYC pathway) and highlighting the pivotal role of miR-155 in regulating glucose metabolism in breast cancer [90]. Although miR-155 has been considered as a potential noninvasive molecular marker for breast cancer screening, it is important to clarify that further research is needed to fully establish its effectiveness [92].

### 5.3 Mitochondrial microRNAs (mitomiRs)

While miRNAs primarily function in the cytoplasm, some studies have suggested the presence of a subset of microRNAs within the mitochondria. These miRNAs, called “mitomiRs” can regulate mitochondrial homeostasis, mtDNA maintenance, and Ca^2+^ balance and therefore play a major role in cancer metabolic reprogramming [93]. The mitomiRs that have been identified in TNBC include miR-29a, miR-140, let-7a, miR-128, miR-30a as glucose metabolism regulators and miR-4485 as an OXPHOS metabolism regulator [94]. The field of mitochondrial miRNA research is dynamically evolving, underscoring ongoing efforts to unravel precise functions and mechanisms.

## 6. Mitochondria: Metabolism and Calcium

Mitochondria, as dynamic organelles, actively participate in a multitude of vital physiological processes. Beyond their primary function of ATP production, they are involved in the regulation of essential cellular processes such as programmed cell death, generating ROS, and certain biosynthetic intermediates needed for metabolic and signaling pathways [95,96]. Mitochondria are also able to sense and uptake cellular calcium, which is correlated with mitochondrial ATP production and regulating various metabolic enzymes, adding to their catalog of contributions on cancer metabolism [50,97–100]. The divergence of cancer cells using OXPHOS as an energy factory is a favorable adaptation that supports the allocation of structural intermediates for sustained survival, especially in advanced and invasive cancers, such as TNBC [7,101,102].

### 6.1 Oxidative Phosphorylation (OXPHOS)

A study that characterized the metabolic profile of breast cancer cells, *in vitro*, revealed that among their panel of cells, TNBC cells presented elevated glycolysis and reduced OXPHOS rates compared to other types of receptor positive breast cancer cells [103]. Another study supplemented these findings with tumor tissues gene expression data, reporting that OXPHOS complex subunit genes were downregulated in TNBC tumor tissue samples compared with non-TNBC tissues [47]. A byproduct of OXPHOS is ROS and interestingly, TNBC cells display higher levels of ROS compared with other subtypes of breast cancer [104]. Excess mitochondrial ROS can trigger apoptotic signals in the cell, but a moderate amount of ROS can be used as a potent signaling molecule in order to adapt to the stress of the TME, especially when a cancer becomes more advanced [50,98,101,102,105,106]. For instance, the transcription factor HIF-1*α* utilizes ROS as a signaling molecule to maintain the transcription of genes favoring metabolic plasticity and angiogenesis [96,101,102,107]. One of the numerous ways that cancer cells maintain their redox state and mitigate the negative effects of excess ROS is by generating nicotinamide adenine dinucleotide phosphate (NADPH) to act as an antioxidant. This helps the cells avoid apoptosis or necrosis, as well as, providing a nucleotide backbone for continued assembly of cellular biomass [96,101].

### 6.2 Mitochondrial Calcium and Mitochondrial Enzymes

Advanced cancers can also avoid unwanted cell fate and maintain survival by modulating their calcium signaling. Calcium ions (Ca^2+^) are secondary messengers which regulate a variety of cellular functions. The frequency, duration, and volume of Ca^2+^ concentrations in the cell, elicits a diverse cellular response, ranging from cell death to cell migration, activation of specific transcription factors and stimulating metabolism, processes that attribute to the aggressiveness of certain cancers [105,108]. There are many different types of Ca^2+^ channels, transporters, and regulatory proteins involved in facilitating the movement of Ca^2+^ in the extracellular and intracellular space, making their way through organelles such as the ER and mitochondria [97,108–110]. Here we focus on mitochondrial Ca^2+^ as it contributes to the regulation of metabolism and the invasiveness of TNBC. Since cancer cells rely on the production of metabolic intermediates, such as amino acids precursors, nucleotides, lipids, and sugars over mitochondrial ATP production, they become highly dependent on glycolysis and the tricarboxylic acid (TCA) cycle for the generation of these intermediates (among other pathways). In this way, cancer cells reprogram their metabolism to tightly regulate the amount of substrate that is produced in the TCA cycle and thereby fed into OXPHOS. There are four TCA cycle dehydrogenase enzymes that are sensitive to mitochondrial Ca^2+^ concentrations for regulating their enzymatic activity and substrate availability. Mitochondrial glycerol-3-phosphate dehydrogenase (GPDH), oxoglutarate (also known as *α*-ketoglutarate) dehydrogenase (OGDH), and isocitrate dehydrogenase (IDH) utilize Ca^2+^ directly for their enzymatic activity [99,111,112]. Pyruvate dehydrogenase (PDH), on the other hand, indirectly relies on its activation through dephosphorylation by the Ca^2+^ sensitive pyruvate dehydrogenase phosphatase (PDP) [99,111,112]. Stimulation of IDH and OGDH enzymatic activity by Ca^2+^ is crucial for OXPHOS to proceed as both enzymes generate NADH that feeds into the respiratory chain (Fig. 2).

### 6.3 Mitochondrial Calcium Uniporter (MCU) and TNBC

At the OMM, mitochondria uptake their calcium through VDAC, mentioned previously for its role in glycolytic cancers and at the inner mitochondrial membrane (IMM) through the mitochondrial calcium uniporter (MCU) [97,113]. Several studies have analyzed *MCU* mRNA expression levels in available cancer patient databases and found that MCU expression is elevated in breast cancer as compared with normal tissues, and certain subtypes such as invasive ductal carcinoma have higher MCU expression when compared with ductal carcinoma [114–116]. MCU overexpression is also correlated with poor patient prognosis and with lymph node invasion in breast cancer patients [106,114,115]. Notably, when MCU expression was compared between 180 breast cancer patients, it was found that MCU is the most elevated in basal-like (TNBC subtype) molecular type of breast cancer [117]. Interestingly, a majority of the studies that have investigated the role of MCU in breast cancer migration and invasion have used TNBC cell lines to address these questions due to the aggressive nature of this cancer [49,106,114,115,117–120]. One study looking at three different TNBC cell lines, MDA-MB-231, MDA-MB-468, and BT-549, showed that siRNA silencing of MCU reduced cell migration in all three TNBC cell lines. Furthermore, in MCU deleted MDA-MB-231 mouse xenografts, they observed a significant reduction in tumor progression and lymph node infiltration [106]. Additionally, the same study found that MCU silencing reduced ROS production, HIF-1*α* transcription, and cell migration [106]. It is crucial to note the complexity of the metabolic fluxes involved in TNBC that go beyond glycolysis and OXPHOS, especially in conjunction with the intricacy of mitochondrial Ca^2+^. A recent *in vitro* study showed TNBC cells having sustained IP3 Ca^2+^ oscillations that are linked to their mitochondrial Ca^2+^ uptake. This was shown to be linked to regulating fatty acid oxidation and reducing cell migration in TNBC cells [118]. Further research contributions that intersect the topics of metabolic reprogramming and mitochondrial Ca^2+^ in difficult to treat cancers such as TNBC can create novel opportunities for therapeutic targets.

## 7. Therapeutic Approaches Targeting Cancer Metabolic Reprogramming

### 7.1 Glycolytic Inhibitors

Advanced cancer diagnoses are often met with limited treatment options. Unlike other subtypes of breast cancer, patients with TNBC pose unique challenges for practitioners due to the lack of target receptors, therefore, targeting metabolic reprogramming in this cancer has emerged as a novel avenue. Some of these novel approaches, include the combination of metabolic inhibitors with current standard treatments, chemotherapy, radiation therapy, and immunotherapy, to improve outcomes for TNBC patients. An innovative approach utilizes nanoparticles for targeted drug delivery in cancer patients, including those with TNBC, to enhance treatment effectiveness while minimizing side effects and dosage. For example, nanoparticles have been employed as dual-delivery systems for combining chemotherapy agents with metabolic inhibitors. Some groups have investigated nanoparticles to deliver paclitaxel, a chemotherapy agent, and lonidamine, a glycolytic inhibitor to cancer cells, aiming to counter the Warburg effect and reduce the highly glycolytic phenotype of certain cancers [121,122]. Another possible avenue involves diclofenac, a non-steroidal anti-inflammatory drug which reduced cell proliferation and increased apoptosis, particularly in TNBC by uniquely targeting c-MYC and thereby reducing glucose metabolism and lactate transport [123]. Pilot clinical trials have been exploring dietary interventions such as ketogenic diets and controlled fasting as well as the use of natural compounds in conjunction with chemotherapy and radiation treatment for advanced cancers [124–127]. Some of these pilot studies have suggested that a ketogenic diet alongside chemotherapy could potentially yield better results for specific breast cancer subtypes. However, it is crucial to emphasize that there is currently no conclusive evidence regarding the effectiveness of this approach, and more extensive research and large-scale clinical trials are necessary to assess both its safety and efficacy [128].

### 7.2 Targeting MCU

Researchers have begun investigating a way to target MCU, as a result of the increased interest in mitochondrial Ca^2+^ and the correlation between TNBC and MCU overexpression [129–131]. In fact, 1600 FDA-approved drugs were screened for their ability to modulate mitochondrial Ca^2+^ without affecting cytosolic Ca^2+^ [129]. This research group identified a negative modulator of MCU, benzethonium, an antiseptic, which inhibited cell growth and migration in TNBC. Interestingly, another group synthesized an analog of the flavonoid protoapigenone, RY10–4. This drug was selectively cytotoxic to cancer cells and caused an increase in the expression of MCU which led to Ca^2+^ overload and in turn cell death in TNBC cells [130]. Studies like these are relatively recent and shed light on the intricacy and autonomy that can exist between cancers and even within the same type of cancer. Recognizing the molecular and clinical heterogeneity within TNBC subtypes is crucial, as therapeutic approaches may vary in effectiveness among them. Tailoring metabolic reprogramming targeting to different molecular subtypes within TNBC is key. Using cancer metabolism as a therapeutic target holds promise for enhancing cancer treatments significantly. For a deeper understanding of this growing field, we invite readers to consult recent articles on metabolic therapies [132,133].

## 8. Conclusions

TNBC is a challenging subtype of breast cancer characterized by its lack of targetable receptors and aggressive nature. This review explored some of the mechanisms behind the complexity that underlies the metabolic reprogramming of this invasive breast cancer. Metabolic reprogramming in TNBC involves an increased reliance on glycolysis, influenced by oncogenic signaling pathways and mRNA regulation by microRNAs. Mitochondria once believed to be impaired, have now been clearly demonstrated to play an integral role in the metabolic adaptations observed in TNBC. Therapeutic approaches are advancing, including the use of metabolic inhibitors, targeted nanoparticles, and innovative drugs. Compounds targeting mitochondrial calcium uptake are emerging as potential candidates for treatment. This evolving understanding of cancer metabolism offers hope for improved therapeutic strategies and approaches to ultimately improve patient outcomes. As we continue to unravel the complexities of cancer biology and metabolic pathways, we are on the edge of significant breakthroughs that could significantly enhance patient outcomes and eventually revolutionize the field of cancer care.

## Figures and Tables

**Table 1.** The Glycolysis and OXPHOS Status in Different Types of Breast Cancer and Their Invasiveness.

Type of breast cancer	Invasiveness	Glycolysis	OXPHOS	Ref.
HER 2+ (i.e., *SKBR3, MDA-MB-453*)	Moderately invasive	Medium	Medium	[25]
TNBC (i.e., *MDA-MB-231, Hs578T*)	Very invasive	Up	Down/Medium	[25,26]
*Breast cancer stem cells (BCSCs)	Very invasive	Up	Up	[27,28]
Inflammatory breast cancer IBC TN-IBC (i.e., *SUM-149*)	Extremely invasive	Up	Up	[29]
Endocrine resistant (i.e., *MCF7 Tamoxifen Resistant*)	Invasive	Up	Down	[30]

“Up” “Down” and “Medium” in the glycolysis and oxidative phosphorylation (OXPHOS) columns represent the status of these metabolic pathways compared to non-invasive, estrogen receptor (ER)+ breast cancer cells as Luminal A.

* Breast cancer stem cells (BCSCs) subpopulation of heterogeneous breast cancer cells demonstrating strong self-renewal and generally identified by the expression of the surface markers CD44, CD24 (Cluster of Differentiation 44 and 24) and ALDH1 (Aldehyde Dehydrogenase 1+). HER2, Human epidermal growth factor receptor 2; TNBC, Triple-negative breast cancer; BCSCs, Breast cancer stem cells; IBC, Inflammatory breast cancer; TN-IBC, Triple-negative subtype of IBC.
